# Evaluating Laparoscopic and Robotic Liver Resection in Elderly Patients: A NSQIP Analysis of Short‐Term Outcomes

**DOI:** 10.1002/jso.70065

**Published:** 2025-08-14

**Authors:** Alessandro Parente, Kevin Verhoeff, Mohamed Elmasry, Blaire L. Anderson, Khaled Z. Dajani, Parthi Srinivasan, A. M. James Shapiro, Krishna V. Menon

**Affiliations:** ^1^ King's College Hospital NHS Foundation Trust Institute of Liver Studies London UK; ^2^ Faculty of Life Sciences and Medicine, School of Immunology and Microbial Sciences, The Roger Williams Institute of Liver Studies King's College London London UK; ^3^ Division of Transplantation, Department of Surgery University of Alberta Edmonton Alberta Canada

**Keywords:** elderly, hepatectomy, laparoscopic hepatectomy, laparoscopic liver resection, robotic hepatectomy, robotic liver resection

## Abstract

**Introduction:**

Results of minimally invasive laparoscopic (LLR) and robotic liver resection (RLR) have been promising, but the benefits in the elderly patients are still unclear. This study aims to compare short‐term outcomes of LLR and RLR in elderly patients.

**Methods:**

The 2017–2021 NSQIP database was analyzed comparing patients ≥ 65 years old undergoing LLR versus RLR. Postoperative outcomes, factors associated with complications and mortality were assessed using propensity score matched (PSM) and multivariable logistic regression.

**Results:**

We analyzed 2,210 patients undergoing LLR (*n* = 1865,84.4%) and RLR (*n* = 345,15.6%). Patients undergoing LLR were older (72.4 vs. 71.8 years; *p* = 0.04) and more likely to have ASA 4 (11.1% vs. 4.9%; *p* = 0.001). RLR patients had shorter hospital stays (3.5 vs. 4.4 days; *p* < 0.001) but longer operative durations (221.4 vs. 203.5 min; *p* = 0.013). On adjusted analyses, RLR was not associated with increased odds of serious complications (OR: 0.82, CI95% 0.42‐1.58, *p* = 0.545) or mortality (OR: 0.87, *p* = 0.851). After PSM, RLR statistically reduced length of stay (−0.72 days; *p* = 0.012) but increased operative times ( + 32.62 min; *p* < 0.001). Subgroup analysis of patients ≥ 75 years confirmed consistent findings.

**Conclusions:**

RLR provides comparable safety and short‐term outcomes to LLR, offering shorter hospital stays but longer operative durations. Findings support RLR as a viable option in elderly patients, but further studies evaluating long‐term outcomes are warranted.

## 1 Introduction

Liver resection (LR) is a well‐established treatment for both primary and metastatic liver lesion. As a result of population ageing, the number of elderly patients requiring surgeries, and thus LR, is steadily increasing. In this particular group, beyond the specific LR risks, previous studies evaluating different abdominal surgeries have also shown that Postoperative morbidity and mortality increases with age [1, 2, 3, 4]. Lately, minimally invasive surgery for LR (MILR) has garnered worldwide interest. The two different minimally invasive approaches, namely laparoscopic and robotic, have been shown to reduce rates of serious complications and length of hospital stay (LOS), without compromising oncological outcomes [5, 6, 7]. Some studies have compared laparoscopic liver resection (LLR) to robotic liver resection (RLR) based on the anatomical sites of the tumor location and extent of the resection [5, 6, 7]. They have shown that RLR might facilitate textbook outcomes and, for difficult segments, RLR was associated with superior perioperative outcomes in terms of decreased operative time, blood loss, and open conversion rate when compared with LLR [5, 6, 7],

However, as the number of reports following MILR continues to rise, it appears important to analyze the outcomes in the elderly population. We aimed to evaluate demographic and short‐term outcome between elderly patients receiving minimally invasive surgery comparing laparoscopic and robotic approaches using the National Surgical Quality Improvement Program (NSQIP) database.

## 2 Methods

### 2.1 Data Source

The NSQIP hepatic‐targeted participant use data, which collects LR specific outcomes, and general database for years 2017–2021 were merged. The NSQIP database collects data across 874 hospitals in Canada and the United States and assembles information from the Preoperative period and up to 30‐days postoperatively for patients undergoing surgery. NSQIP data is collected by trained surgical clinical reviewers and is deidentified to hospital, patient, surgeon, and region. In view of this, this study was exempt from ethics approval.

### 2.2 Study Design

We performed a retrospective analysis of prospectively collected NSQIP data. We identified patients undergoing any hepatectomy using current procedural terminology (CPT) codes 47120 (hepatectomy, partial lobectomy), 47125 (left lobectomy), 47130 (right lobectomy), and 47122 (trisegmentectomy). For this study, only patients aged ≥ 65 years old were included based on the definition of the World Health Organization for elderly person.

### 2.3 Study Population and Objectives

The primary objective of this study was to compare LLR and RLR in elderly patients ( ≥ 65 years old) regarding demographics and early Postoperative 30‐day outcomes. All demographics and outcomes are described according to NSQIP predetermined definitions [8]. Open LR cases were excluded. Firstly, patients were characterized based on the type of surgery (partial lobectomy, right lobectomy, left lobectomy, or trisegmentectomy) and whether patients received biliary reconstruction (i.e., hepaticojejunostomy) or not. Looking at the demographics, age, sex, body mass index (BMI), and aggregate measures of comorbidity including functional status before surgery (independent, partially dependent, unknown) and their American Society of Anesthesiologists (ASA) score were compared. Specific comorbidities were also analyzed and included cardiac conditions such as congestive heart failure (CHF) and hypertension. Pulmonary comorbidities evaluated included chronic obstructive pulmonary disease (COPD), and active smoking status. Other comorbidities evaluated in this study included diabetes, chronic steroid use, kidney failure on dialysis treatment, and diagnosis of Preoperative bleeding disorder. Oncologic factors characterized in this study were Preoperative neoadjuvant therapy, tumor size, and tumor invasion (described as T3 or T4 tumor status). Lastly, Preoperative sepsis (none, systemic inflammatory response syndrome, sepsis) was also illustrated.

### 2.4 Study Definitions

The evaluation of postoperative outcomes was based on NSQIP predetermined definitions [8]. Herein, we analyzed operative time, LOS, discharge destination (home, rehabilitation, increased level of care), unplanned reoperations, readmission to hospital within 30 days, and if the patients were still admitted beyond 30 days following surgery. Infectious Postoperative complications were extracted including wound complications (superficial surgical site infection (SSI), deep SSI, organ space SSI, wound disruption/dehiscence), pneumonia, and urinary tract infection. Moreover, we also collated information regarding specific post‐LR complications, namely bile leak, including any bile leak requiring drain maintenance, percutaneous drain placement, or reoperation. We also characterized any post hepatectomy liver failure and graded these complications according to the International Study Group of Liver Surgery [9]. Postoperative bleeding was defined as any bleeding requiring ≥ 1 unit blood transfusion. Serious complications included respiratory complications (unplanned intubation, pulmonary embolism), cardiovascular complications (myocardial infarction, cardiac arrest), acute renal failure, sepsis, septic shock, deep vein thrombosis (DVT), and cerebral vascular accidents. An aggregate measure of serious complications was also evaluated. This was defined as the presence of ≥ 1 serious complication as detailed above, reoperation, still in hospital > 30 days, bile leak requiring percutaneous or operative intervention, Grade B and C post‐hepatectomy liver failure, or mortality within 30 days of operation. Additionally, the Clavien Dindo Classification (CDC) [10] and the updated Comprehensive Complications Index (CCI) were utilized to evaluated Postoperative complications [11], as CCI has shown to be a superior measure of complications and associated with oncologic outcomes [12, 13]. To calculate CCI, the Postoperative complications were assessed and treatments were assumed based on accepted treatments for these complications as described in Supporting Information Table S1.

### 2.5 Statistical Analysis

Data in this study were assumed to be normally distributed due to large sample sizes and as such, continuous variables are presented as mean ± standard deviation with cohort differences evaluated using regression. Counts and percentages are presented for categorical variables and analyzed for differences with Chi squared tests. The alpha was set as *p* < 0.05.

To control for demographic differences and adjust for cohort size mismatch multivariable logistic modelling was performed to investigate factors (including LLR and RLR operative approach) independently associated with serious complications and mortality. Models were established using a hypothesis driven purposeful methodology ‐ univariate analysis was performed to investigate the association between variables in Table 1 and serious complications and mortality. Factors with a *p* value < 0.10 and clinically relevant factors based on previous studies were then used to generate a preliminary main effects logistic regression model, which was evaluated for collinearity using the variance inflation factor, with values > 10 prompting exclusion from the model. A final model was then produced and assessed for goodness of fit using the Brier Score (BS) and receiver operating characteristic (ROC) curves.

**Table 1:** Demographics of patients ≥ 65 years old undergoing liver resection comparing those receiving laparoscopic and robotic approaches.

	Laparoscopic *n* = 1,865 *n* (%)	Robotic *n* = 345 *n* (%)	*p* value*
Surgery performed			0.460
Liver wedge resection	1,600 (85.8)	295 (85.5)	
Left hepatectomy	116 (6.2)	21 (6.1)	
Right hepatectomy	96 (5.2)	23 (6.7)	
Trisegmentectomy	53 (2.8)	6 (1.7)	
Biliary reconstruction	47 (2.5)	4 (1.2)	0.122
Age, years (mean ± sd)	72.4 (5.6)	71.8 (5.7)	***0.040***
Female Sex	799 (42.8)	174 (50.4)	***0.009***
BMI in Kg/m^2^ (mean ± sd) Kg/m^2^	27.9 (5.7)	28.5 (5.4)	0.052
Functional status			0.120
Independent	1,832 (98.2))	344 (99.7)	
Partially dependent	28 (1.5)	1 (0.3)	
Totally dependent	5 (0.3)	(0.0)	
ASA Category			***0.001***
1	4 (0.2)	2 (0.6)	
2	354 (19.0)	56 (16.2)	
3	1,299 (69.7)	270 (78.3)	
4	206 (11.1)	17 (4.9)	
5	0 (0.0)	0 (0.0)	
Smoker	202 (10.8)	50 (14.5) .88)	***0.049***
Diabetes			
No or diet controlled	1,382 (74.1)	259 (75.1)	0.422
Non‐insulin dependent	326 (17.5)	64 (18.6)	
Insulin dependent	157 (8.4)	22 (6.4)	
Severe COPD	108 (5.8)	15 (4.4)	0.283
CHF	13 (0.7)	2 (0.6)	0.973
Hypertension	1,219 (65.4)	245 (71.0)	***0.040***
Sepsis			
None	1,964 (98.6)	676 (99.0)	
SIRS	10 (0.5)	0 (0.0)	0.270
Sepsis	10 (0.5)	0 (0.0)	
Dialysis dependent	13 (0.7)	2 (0.6)	0.807
Chronic steroid use	56 (3.0)	15 (4.4)	0.193
Bleeding disorder	87 (4.7)	18 (5.2)	0.658
Weight Loss > 10% monthss	39 (2.5)	5 (1.9)	0.560
Neoadjuvant	316 (17.0)	62 (18.1)	0.611
Invasion	86 (10.5)	17 (12.3)	0.514
Tumor size			0.546
< 2 cm	234 (12.6)	46 (13.3)	
2‐5 cm	370 (19.8)	79 (22.9)	
> 5 cm	220 (11.8)	38 (11.0)	
Not reported	1,041 (55.8)	182 (52.8)	

*Note:* Bold values indicate statistically significant.

Abbreviations: ASA, American Society of Anesthesiologists; BMI, body mass index; COPD, chronic obstructive pulmonary disease.

Although multivariable modelling is statistically superior with large datasets, a propensity score matched (PSM) analysis was completed due to the relatively small number of patients undergoing RLR and to better assess outcomes including mortality, length of stay, and aggregate complication measures. For PSM, patients undergoing LLR were matched to those undergoing RLR using probit treatment models controlling for age, BMI, diabetes status, type of surgery, and whether patients received bile duct reconstruction. Replacements were allowed to improve the matching accuracy, meaning LLR patients could match more than once to RLR patients. Results are provided as the mean difference with 95% confidence intervals (95CI) between LLR and RLR cohorts using an average estimate of treatment effect in the treated analysis. All analyses were conducted using Stata 17 (STATACorp LP, College Station, TX).

## 3 Results

### 3.1 Baseline Characteristics

This study analyzed 2,210 patients undergoing LR of whom 1865 (84.4%) and 345 (15.6%) receiving LLR and RLR, respectively. Patients undergoing LLR were older (72.4 vs. 71.8 years; *p* = 0.04) and less likely to be female (*p* = 0.009) and had a higher ASA 4 score (11.1% vs. 4.9%; *p* = 0.001). Preoperative hypertension and smoking were more frequent in the RLR group, but other comorbidities, demographics, and tumor characteristics were comparable (Table 1).

### 3.2 Postoperative Outcomes

Unadjusted analysis of postoperative outcomes at 30 days demonstrated that patients undergoing LLR had a shorter operative duration (203.5 vs. 221.4 min; *p* = 0.013), however the RLR group had a significantly shorter LOS (3.5 vs. 4.4 days; *p* < 0.001; Table 2). Patients undergoing RLR had a lower rate of myocardial infarction (0.3% vs 1.7%, *p* = 0.045), postoperative liver failure (3.1% vs. 7.8%, *p* < 0.001) and superficial SSI (0.6% vs 2.3%, *p* = 0.037), but had higher rates of septic shock (1.7% vs 0.6%, *p* = 0.038). The most common CDC complication was grade 2 in both groups; however, no statistical differences were found in the rates of CDC and overall CCI scores. There were no significant differences in postoperative strokes, MI, or mortality (Table 2).

**Table 2:** Thirty‐day postoperative outcomes for patients ≥ 65 years old undergoing liver resection comparing those receiving laparoscopic and robotic approaches.

	Laparoscopic *n* = 1,865 *n* (%)	Robotic *n* = 345 *n* (%)	*p* value
Operative duration (minutes; mean ± SD)	203.5 (123.6)	221.4 (116.4)	***0.013***
Length of stay (days; mean ± SD)	4.4 (4.5)	3.5 (2.9)	***< 0.001***
Unplanned reoperation	32 (1.7)	10 (2.9)	0.139
Discharge destination			
Home	1,749 (93.8)	324 (93.9)	
Rehab	31 (1.7)	2 (0.6)	***0.040***
Skilled care, not home	36 (1.9)	8 (2.3)	
Other	49 (2.6)	11 (3.2)	
Still in hospital > 30 days	17 (0.9)	1 (0.3)	0.238
Readmission	131 (7.0)	28 (8.1)	0.471
Superficial SSI	43 (2.3)	2 (0.6)	***0.037***
Deep SSI	3 (0.2)	1 (0.3)	0.605
Organ Space SSI	70 (3.8)	15 (4.4)	0.598
Wound disruption	3 (0.2)	1 (0.3)	0.605
Sepsis	38 (2.0)	2 (0.6)	0.062
Septic shock	12 (0.6)	6 (1.7)	***0.038***
Pneumonia	50 (2.7)	5 (1.5)	0.177
Unplanned intubation	34 (1.8)	7 (2.0)	0.795
DVT	14 (0.8)	4 (1.2)	0.438
Pulmonary embolism	14 (0.8)	3 (0.9)	0.816
Acute renal failure	15 (0.8)	4 (1.2)	0.512
Urinary tract infection	33 (1.8)	3 (0.9)	0.255
Cerebral vascular accidents	5 (0.3)	1 (0.3)	0.943
Myocardial infarction	32 (1.7)	1 (0.3)	***0.045***
Cardiac arrest	10 (0.5)	2 (0.6)	0.920
Liver failure grade			
A	24 (1.3)	2 (0.6)	
B	15 (0.8)	1 (0.3)	0.372
C	13 (0.7)	4 (1.2)	
Liver failure (any grade)	156 (7.8)	21 (3.1)	***< 0.001***
Bleed	186 (10.0)	38 (11.0)	0.556
Serious complication	159 (8.5)	24 (7.0)	0.331
Comprehensive complication index (mean ± SD)	6.6 (14.3)	6.6 (14.6)	0.990
Clavien dindo complications			0.283
1	75 (4.0)	11 (3.2)	
2	241 (13.0)	46 (13.4)	
3 A	26 (1.4)	6 (1.7)	
3B	21 (1.1)	5 (1.5)	
4 A	53 (2.9)	3 (0.9)	
4B	21 (1.1)	7 (2.0)	
Mortality	29 (1.6)	5 (1.5)	0.884

*Note:* Bold values indicate statistically significant.

Abbreviations: MI, myocardial infarction; POPF, post‐operativepostoperative pancreatic fistula; SSI, surgical site infection; VTE, venous thromboembolism.

### 3.3 Predicting Serious Complications and Mortality After Controlling for Demographic and Surgical Differences

Following demographic difference adjustment with multivariable logistic modelling a RLR approach compared to LLR was not associated with a significantly lower odds of serious complications (OR:0.82, CI 95% 0.42–1.58, *p* = 0.545; Table 3). These results were achieved after controlling for surgery type, whereby undergoing right hepatectomy (OR 4.5; *p* < 0.001), but not trisegmentectomy (OR 1.43; *p* = 0.514) and left hepatectomy (OR 1.34; *p* = 0.464), was independently associated with a higher odds of serious complications compared to partial LR (i.e., wedge resection). Notably, biliary reconstruction was associated with serious complications (OR 6.77; *p* < 0.001; Table 3).

**Table 3:** Multivariable logistic regression evaluating predictors of serious complications in patients ≥ 65 years old undergoing minimally invasive liver resection.

Risk factor	Odds ratio	95% Confidence interval	*p* value
Robotic (compared to laparoscopic)	0.82	0.42–1.58	0.545
Age	1.01	0.97–1.05	0.597
BMI	1.03	0.99–1.07	0.170
Sex	1.11	0.70–1.76	0.659
COPD	0.73	0.27–1.99	0.537
CHF	1.21	0.15–9.71	0.857
HTN	1.00	0.57–1.75	0.994
Diabetes			
Type 1 diabetes	2.37	1.25–4.52	0.008
Type 2 diabetes	1.68	0.98–2.85	0.057
Smoking	1.60	0.86–2.96	0.136
Dialysis dependence	3.56	0.80–15.82	0.095
Preoperative steroid use	1.31	0.37–4.60	0.675
Bleeding disorder	0.45	0.14–1.51	0.199
Preoperative sepsis	3.43	0.43–27.17	0.243
Partially dependent functional status (compared to independent)	2.43	0.74–8.03	0.145
Invasion (T3 or T4)	1.72	0.94–3.15	0.079
Surgery (compared to wedge resection)			
Left hepatectomy	1.34	0.61–2.97	0.464
Right hepatectomy	4.50	2.36–8.57	**< 0.001**
Trisegmentectomy	1.43	0.49–4.16	0.514
Bile duct reconstruction	6.77	2.87–15.96	**< 0.001**

*Note:* Brier score = 0.084. ROC area = 0.704. Bold values indicate statistically significant.

Abbreviations: BMI, body mass index; CHF, congestive heart failure; COPD, chronic obstructive pulmonary disease.

Evaluation of factors independently associated with mortality demonstrated that RLR approach was not associated (OR: 0.87, CI95%0.21–3.60, *p* = 0.851; Table 4) with any mortality difference when compared to LLR. Similar to complications, right hepatectomy was associated with increased odds of mortality (OR 5.15; *p* < 0.001), while left hepatectomy (OR 3.29; 0.091) and trisegmentectomy (OR 5.43; *p* = 0.098) were not. Conversely, receiving biliary reconstruction was not linked with mortality (OR 1.35; *p* = 0.768). Looking at the preoperative comorbidities, type 1 diabetes, preoperative sepsis, and partially dependent functional status were also associated with increased mortality (Table 4).

**Table 4:** Multivariable logistic regression evaluating predictors of mortality in patients ≥ 65 years old undergoing minimally invasive liver resection.

Risk factor	Odds ratio	95% Confidence interval	*p* value
Robotic (compared to laparoscopic)	0.87	0.21–3.60	0.851
Age	1.02	0.94–1.13	0.504
BMI	1.07	0.99–1.15	0.064
Sex	0.46	0.15–1.39	0.169
COPD	0.23	0.01–3.89	0.310
CHF	3.58	0.18–69.61	0.400
HTN	0.61	0.19–1.97	0.409
Diabetes			
Type 1 diabetes	6.92	2.32–20.61	**0.001**
Type 2 diabetes	0.93	0.24–3.60	0.913
Smoking	1.14	0.29–4.53	0.850
Dialysis dependence	—	—	—
Preoperative steroid use	—	—	—
Bleeding disorder	1.11	0.20–6.18	0.906
Preoperative sepsis	40.81	3.91–425.47	**0.002**
(compared to independent)	5.86	1.10–31.30	**0.039**
Neoadjuvant therapy	0.66	0.07–6.44	0.718
Invasion (T3 or T4)	2.30	0.75–7.08	0.145
Surgery (compared to wedge resection)			
Left hepatectomy	3.29	0.83–13.05	0.091
Right hepatectomy	5.15	1.42–18.71	**0.013**
Trisegmentectomy	5.43	0.73–40.31	0.098
Bile duct reconstruction	1.35	0.18–9.92	0.768

*Note:* Brier score = 0.026. ROC area = 0.802. Dialysis dependence and preoperative steroid use were excluded as they predicted failure perfectly. Bold values indicate statistically significant.

Abbreviations: BMI, body mass index; CHF, congestive heart failure; COPD, chronic obstructive pulmonary disease.

Factors included in PSM included age, BMI, diabetes status, type of surgery, and whether patients received bile duct reconstruction (Table 5). Following PSM, assessment of outcomes demonstrated a significant reduction in LOS (−0.72 days; 95CI −1.29% to −0.16; *p* = 0.0124) with RLR and no difference in mortality. However, after PSM RLR was noted to have a significantly longer operative duration (32.62 min; 95% CI 15.82 to 49.41 min; *p* < 0.001).

**Table 5:** Propensity matched analysis of thirty‐day postoperative outcomes for patients ≥ 65 years old undergoing liver resection comparing laparoscopic and robotic surgical approaches.

Outcome	ATE	95% CI	*p* value
Serious complications	0.90%	−3.04 to 4.79	0.664
Mortality	0.30%	−1.50 to 2.10	0.750
Length of stay (days)	−0.72	−1.29 to −0.16	0.012
Comprehensive complication index	1.42	−0.68 to 3.53	0.186
Clavien‐dindo classification complication grade	0.004	−0.19 to 0.20	0.965
Operation time	32.62	15.82 to 49.41	< 0.001

*Note:* ATE, average treatment effect; 95% CI, 95% confidence interval.

### 3.4 Subgroup Analysis of Patients Aged 65–75 and ≥ 75 Years Old

Additional adjusted analyses for patients aged 65–74 and ≥ 75 years old were performed, including multivariable logistic regression evaluating serious complications and propensity matched analysis performed as described above. Analysis of patients aged 65–74 included 1,527 patients (1,276 LLR vs. 251 RLR). Multivariable logistic regression demonstrated that in patients aged 65‐74, RLR was not independently associated with serious complications (OR 0.84, *p* = 0.666; Supporting Information Table S2). Propensity matched analysis matching only patients aged 65–74 on age, BMI, diabetes, and type of surgery (left hepatectomy, right hepatectomy, trisegmentectomy, or partial hepatectomy) demonstrated a trend towards shorter length of hospital stay (−0.57 days; 95% CI −1.18 to 0.04 days; *p* = 0.069) and a significantly longer operative duration (24.90 min; 95% CI 5.31 to 44.49 min; *p* = 0.013) with RLR compared to LLR. Other outcomes were similar between cohorts (Supporting Information Table S3).

Analysis of patients ≥ 75 included 683 patients, with 589 receiving LLR and 94 undergoing RLR. Similar to the entire cohort and the middle‐aged cohort, in patients ≥ 75 there was no independent effect of RLR (compared to LLR) on serious complications (OR 0.75, *p* = 0.646; Supporting Information Table S4). Additionally, following propensity matched analysis of only patients ≥ 75 years old comparing LLR and RLR, no differences in any outcomes were noted (Supporting Information Table S5).

## 4 Discussion

This study presents the analysis of a large population group of elderly patients ≥ 65 years old comparing short‐term outcomes of LLR and RLR. Both MILR approaches displayed satisfactory outcomes with similar rates of postoperative CDC and CCI. However, elderly patients undergoing RLR had a reduction of the overall LOS by almost 1 day. The analysis also identified right hepatectomy (H5678) and biliary reconstruction as independent factors associated with serious complications in MILR. Subgroup analysis of patients aged ≥ 75 years corroborated the main findings. This consistency across age groups underscores the safety of robotic surgery in elderly populations, who may otherwise be at increased risk for adverse outcomes. As the global population ages, the role of minimally invasive techniques in improving recovery and reducing hospital stays for older patients becomes increasingly relevant. These findings provide valuable insights into the evolving role of robotic surgery in hepatobiliary procedures.

In the past decades, MILR has gained popularity due to its advantages compared to open surgery [14, 15]. The steep learning curve for MILR has hampered its rapid widespread, but there is mounting data demonstrating that it can be safely performed in expert centers. Moreover, some studies showed that the robotic platform may facilitate complex procedures, potentially shortening the learning curve, especially for difficult segments or for major LRs [16, 17, 18, 19, 20, 21, 22]. Indeed, the robotic approach has several advantages including improved surgeon's precision by the ten‐fold higher magnification and enhanced vision through 3D images. Additionally, the robotic platform eliminates a surgeon's natural tremor and allows a broader degree of instrument motion leading to better accuracy in many abdominal surgeries [18, 19]. However, the lack of haptic feedback, the limited availability of the platform, relative high costs and the absence of some energy devices, especially cavitron ultrasonic surgical aspirator, represent some known disadvantages compared to the more commonly used laparoscopic approach. In fact, the comparable safety profiles of RLR and LLR highlight the importance of individualizing surgical approaches based on patient characteristics, surgeon expertise, and institutional resources. While RLR offers certain benefits, its longer operative duration and higher upfront costs may limit widespread adoption, particularly in low‐resource settings.

Whilst increased age does not represent an absolute contraindication to major surgical procedures, it has been linked to worse surgical outcomes in various abdominal surgeries, including LR [4, 23, 24]. However, technological advantages, optimization of patients care, and surgical innovation seem to mitigate this age effect with satisfactory postoperative outcomes even in major LR [25, 26, 27, 28]. Some might argue that 65 years old based on World Health Organization is a low threshold to define elderly patients, as it has been suggested that age from 65 to 74 years should be defined as “pre‐old age”, whereas age over 75 years as “old age” [27]. This could be particularly relevant in some countries with longer life expectancy, such as Japan [27]. However, these differences were well‐captured by our subgroup analysis of patients ≥ 75 years old, whereby RLR has also demonstrated to be safe and not linked with increased rates of postoperative complications or worse short‐term outcomes. These results are in line with previous reported studies, where RLR has been linked with excellent outcomes in older patients including major LR [26, 27, 28]. Our study is the first to report a shorter LOS with RLR compared to LLS in elderly patients, however, a benefit in LOS following RLR was not showed in age ≥ 75 years old due to the relatively lower number of patients, which did not allow meaningful analyses, therefore results warrant caution interpretation in this specific subgroup of patients. Additionally, the LOS reduction in our study represents a < 1 day difference, and the clinical significance of this difference is arguable therefore the results of this study regarding LOS should be interpreted with caution.

A large study compared 10.075 patients undergoing either RLR (*n* = 1.507) or LLR (*n* = 8.568) in various settings [7]. The authors demonstrated RLR was associated with lower rates and duration of Pringle maneuver, less blood loss, transfusions, and open conversions. Patients undergoing RLR had lower postoperative morbidity rates but were more often readmitted. In addition, in the RLR group there were higher rates of textbook outcomes, namely absence of intraoperative incidents of grade 2 or higher, postoperative bile leak grade B or C, severe morbidity, readmission, and 90‐day or in‐hospital mortality with the presence of an R0 resection margin in case of malignancy. Our data showed a significant reduction in the LOS with RLR and overall similar postoperative outcomes and rates of CDC and CCI. In fact, there was no significant difference in CDC between groups; however, the LLR group showed higher rates of myocardial infarction, sepsis, and liver failure, and a greater proportion of patients were discharged to rehabilitation facilities. These findings suggest that patient background factors may have contributed to the occurrence of such complications, potentially leading to prolonged LOS. Also, the present study only captured elderly patients aged ≥ 65 years, therefore evaluating a distinct population which is known to be frailer and more likely to have preoperative comorbidities. These differences could possibly be explained by the minimally invasive nature of robotic surgery which may reduce recovery times through enhanced ergonomics and dexterity, allowing for more precise dissection and reduced tissue trauma. Whilst we were not able to show a significant lower overall postoperative comorbidity rate between RLR and LLR, it is plausible that, in line with the previous report [7], RLR necessitated less Pringle maneuvers, minimized blood loss and transfusions which might have led to shorten LOS. Unfortunately, this granular data is not available within NSQIP, thus these reasons can only be speculated. Additionally, it has to be acknowledged that NSQIP only captures data of 30‐days postoperative outcomes by contributing centers based in the US or Canada, possibly owing these differences also to health care policies between the participating centers.

This study has several limitations. Firstly, the retrospective nature of this analysis may be associated with unmeasured confounding factors. Secondly, the NSQIP database does not collect some granular preoperative intraoperative data, and indeed almost half of tumor size or location where not reported making it difficult to define the extent of surgical procedure. Thirdly, data on learning curve is lacking within the NSQIP. RLR is a relatively new approach and some centers contributing to NSQIP might have been at the beginning of the RLR/LLR learning curve, and the lack of this granular data together with the decision‐making processes in patient selection limit further detailed analyses. In fact, the number of RLR cases was relatively small in this cohort, and it is likely that these procedures were performed predominantly at a limited number of high‐volume or expert centers which might have had already established laparoscopic LR programs, therefore creating bias in favoring the positive results towards RLR. As a consequence, expert centers outcomes from MILR approaches may actually be superior to those reported here. This potential selection bias may have influenced the observed differences in outcomes. Additionally, the study did not assess long‐term oncological outcomes, which are critical in evaluating the overall efficacy of LR techniques. Finally, NSQIP data is based on US and Canadian centers, thus limiting the broader applicability of these results in other countries with different population demographics and healthcare systems.

## 5 Conclusions

RLR is safe and feasible in elderly patients, achieving similar short‐term outcomes compared to LLR. However, it appears that the robotic approach might shorten LOS with the caveat of longer operative times. The positive results of the present study encourage the continued integration of RLR into hepatobiliary surgery in elderly patients. Additional long‐term outcomes including oncologic and comparisons in well‐designed randomized studies are warranted to validate our findings.

## Author Contributions

Study concept: Alessandro Parente, Kevin Verhoeff. Study design: all authors. Data curation, investigation and collection: Alessandro Parente, Kevin Verhoeff. Data analysis and interpretation: Alessandro Parente, Kevin Verhoeff. Project administration and resources: Alessandro Parente, Kevin Verhoeff. Resources, validation and visualization: Alessandro Parente, Kevin Verhoeff. Writing – original draft: Alessandro Parente, Kevin Verhoeff, Krishna V. Menon. Writing – review and editing: all authors revised and approved the manuscript.

## Disclosure

American College of Surgeons National Surgical Quality Improvement Program and the hospitals participating in the ACS NSQIP are the source of the data used herein; they have not verified and are not responsible for the statistical validity of the data analysis or the conclusions derived by the authors.

## Conflicts of Interest

The authors declare no conflicts of interest.

## Synopsis

This study evaluated the short‐term outcomes of laparoscopic (LLR) and robotic liver resection (RLR) in patients aged 65 and older using NSQIP data from 2017 to 2021. RLR was associated with shorter hospital stays but longer operative times, with no significant differences in serious complications or mortality compared to LLR. These results support the safety and feasibility of RLR in elderly patients, including those ≥ 75 years, while highlighting the need for further investigation into long‐term outcomes.

## Supporting information

supporting material elderly R1.

## Data Availability

The data that support the findings of this study are available from the corresponding author upon reasonable request. All data from this study is available upon reasonable request to the corresponding author.
