# Supplementary material for: Evaluating Laparoscopic and Robotic Liver Resection in Elderly Patients: A NSQIP Analysis of Short‐Term Outcomes
Source: J Surg Oncol. 2025 Aug 14;132(5):908–16. doi: 10.1002/jso.70065 (PMC12501916; doi:10.1002/jso.70065)
Supplement: Supplementary file 1 — supporting material elderly R1. [file JSO-132-908-s001.docx]

**Table S1.** Complications and their associated Clavien Dindo Classification applied during this study

| **Complication** | **Clavien Dindo Classification** |
| --- | --- |
| Superficial SSI | 1 |
| Deep incision SSI | 1 |
| Readmission | 1 |
| Bile leakage – spontaneous wound drainage | 2 |
| Bile leakage – drain maintained after post-operative day 3 | 2 |
| Liver failure Grade B | 2 |
| Organ space SSI | 2 |
| Pneumonia | 2 |
| Sepsis | 2 |
| DVT requiring therapy | 2 |
| Pulmonary embolism | 2 |
| Transfusion intraoperative or post-operative | 2 |
| Urinary tract infection | 2 |
| Bile leakage requiring percutaneous drain | 3A |
| Wound dehiscence | 3B |
| Return to OR | 3B |
| Cardiac arrest | 4A |
| Myocardial infarction | 4A |
| Acute renal failure | 4A |
| Unplanned intubation | 4A |
| Liver failure Grade C | 4B |
| Septic shock | 4B |
| Mortality | 5 |

**Supplementary Table S2.** Multivariable logistic regression evaluating predictors of serious complications in patients ages 65-74 years old undergoing minimally invasive liver resection

| **Risk Factor** | **Odds Ratio** | **95% confidence interval** | **p-value** |
| --- | --- | --- | --- |
| Robotic (compared to laparoscopic) | 0.836 | 0.37 – 1.89 | 0.666 |
| Age | 0.947 | 0.86 – 1.05 | 0.298 |
| BMI | 1.05 | 0.99 – 1.10 | 0.079 |
| Sex | 1.18 | 0.65 – 2.17 | 0.585 |
| COPD | 1.34 | 0.43 – 4.20 | 0.611 |
| CHF | 0.87 | 0.02 – 43.73 | 0.945 |
| HTN | 1.40 | 0.67 – 2.91 | 0.372 |
| Diabetes |  |  |  |
| Type 1 diabetes | 2.06 | 0.89 – 4.75 | 0.090 |
| Type 2 diabetes | 2.47 | 1.26 – 4.85 | 0.009 |
| Smoking | 2.26 | 1.11 – 4.60 | 0.025 |
| Dialysis dependence | 7.69 | 1.53 – 38.65 | 0.013 |
| Preoperative steroid use | 2.58 | 0.67 – 9.85 | 0.166 |
| Bleeding disorder | 0.22 | 0.03 – 1.81 | 0.160 |
| Preoperative sepsis | - |  |  |
| Partially dependent functional status (compared to independent) | 12.47 | 1.89 – 82.08 | 0.009 |
| Invasion (T3 or T4) | 1.95 | 0.86 – 4.42 | 0.109 |
| Surgery (compared to wedge resection) |  |  |  |
| Left hepatectomy | 1.45 | 0.53 – 3.99 | 0.475 |
| Right hepatectomy | 4.12 | 1.70 – 9.98 | 0.002 |
| Trisegmentectomy | 1.48 | 0.37 – 5.86 | 0.579 |
| Bile duct reconstruction | 7.12 | 2.54 – 19.93 | <0.001 |

Brier score = 0.085

ROC area = 0.679

BMI, body mass index; COPD, chronic obstructive pulmonary disease; CHF, congestive heart failure

**Supplementary Table S3.** Propensity matched analysis of thirty-day post-operative outcomes for patients 65-74 years old undergoing liver resection comparing laparoscopic and robotic surgical approaches. Patients matched for age, BMI, diabetes, and type of surgery (left hepatectomy, right hepatectomy, trisegmentectomy, or partial hepatectomy).

| **Outcome** | **ATE** | **95% CI** | **p-value** |
| --- | --- | --- | --- |
| Serious complications | -1.79% | -6.50 to 2.91 | 0.455 |
| Mortality | -0.40% | -2.32 to 1.53 | 0.685 |
| Length of Stay (days) | -0.57 | -1.18 to 0.43 | 0.069 |
| Comprehensive Complication Index | 1.82 | -4.35 to 0.71 | 0.159 |
| Clavien-Dindo Classification Complication Grade | -0.20 | -0.47 to 0.06 | 0.965 |
| Operation Time | 24.90 | 5.31 to 44.49 | 0.013 |

ATE, average treatment effect; 95% CI, 95% confidence interval

**Supplementary Table S4.** Multivariable logistic regression evaluating predictors of serious complications in patients ≥ 75 years old undergoing minimally invasive liver resection

| **Risk Factor** | **Odds Ratio** | **95% confidence interval** | **p-value** |
| --- | --- | --- | --- |
| Robotic (compared to laparoscopic) | 0.75 | 0.22 – 2.59 | 0.646 |
| Age | 1.07 | 0.96 – 1.19 | 0.237 |
| BMI | 0.97 | 0.90 – 1.05 | 0.508 |
| Sex | 1.12 | 0.49 – 2.57 | 0.784 |
| COPD | 0.15 | 0.01 – 2.05 | 0.155 |
| CHF | 2.97 | 0.19 – 45.75 | 0.436 |
| HTN | 0.53 | 0.20 – 1.41 | 0.202 |
| Diabetes |  |  |  |
| Type 1 diabetes | 5.07 | 1.46 – 17.61 | 0.011 |
| Type 2 diabetes | 1.03 | 0.35 – 3.02 | 0.959 |
| Smoking | 0.88 | 0.16 – 4.78 | 0.885 |
| Dialysis dependence | - | - | - |
| Preoperative steroid use | - | - | - |
| Bleeding disorder | 1.30 | 0.22 – 7.74 | 0.770 |
| Preoperative sepsis | 1.44 | 0.09 – 23.40 | 0.796 |
| Partially dependent functional status (compared to independent) | 0.26 | 0.02 – 3.25 | 0.299 |
| Invasion (T3 or T4) | 1.55 | 0.58 – 4.13 | 0.382 |
| Surgery (compared to wedge resection) |  |  |  |
| Left hepatectomy | 1.36 | 0.32 – 5.75 | 0.676 |
| Right hepatectomy | 9.62 | 3.24 – 28.51 | 0.000 |
| Trisegmentectomy | 0.95 | 0.11 – 8.17 | 0.961 |
| Bile duct reconstruction | 8.94 | 1.13 – 70.93 | 0.038 |

Brier score = 0.090

ROC area = 0.623

BMI, body mass index; COPD, chronic obstructive pulmonary disease; CHF, congestive heart failure

**Supplementary Table S5.** Propensity matched analysis of thirty-day post-operative outcomes for patients ≥ 75 years old undergoing liver resection comparing laparoscopic and robotic surgical approaches. Patients matched for age, BMI, diabetes, and type of surgery (left hepatectomy, right hepatectomy, trisegmentectomy, or partial hepatectomy).

| **Outcome** | **ATE** | **95% CI** | **p-value** |
| --- | --- | --- | --- |
| Serious complications | -4.26% | -12.26 to 3.75 | 0.298 |
| Mortality | 2.13% | -1.66 to 5.91 | 0.270 |
| Length of Stay (days) | -1.09 | -2.36 to 0.19 | 0.094 |
| Comprehensive Complication Index | 0.93 | -2.60 to 4.46 | 0.159 |
| Clavien-Dindo Classification Complication Grade | 0.12 | -0.22 to 0.45 | 0.497 |
| Operation Time | 3.03 | -28.54 to 34.60 | 0.851 |

ATE, average treatment effect; 95% CI, 95% confidence interval
